# Cystinuria

**Published:** 1890-03-08

**Authors:** 


### CYSTINURIA.

L. von Udránski and E. Baumann, working at the so-called ptomaines, have found them constantly present in the urine of persons suffering from cystinuria, though they have examined the urine of cystitis, scarlatina, diphtheria, enteric, pneumonia, peritonitis, and septicæmia in vain. These ptomaines are the Penta and Tetra-methylene diamines $(CH_2)^5 (NH_2)^2$ and $(CH_2)^4 (NH_2)^2$ the so-called cadaverin and putrescin. They consider that cystinuria should be looked on as an infective disease, these bodies being the products of specific microbes, for they were absent in the foulest urine of cystitis, and in cystinuric individuals they were found in the bladder and bowel in inverse proportions.

